# Do Telemedicine Wound Care Specialist Consults Meet the Needs of the Referring Physician? A Survey of Primary Care Providers

**DOI:** 10.1155/2011/321376

**Published:** 2011-08-11

**Authors:** Marek K. Dobke, Dhaval Bhavsar, Fernando Herrera

**Affiliations:** ^1^Division of Plastic Surgery UCSD, Department of Surgery, University of California San Diego, 200 West Arbor Drive, San Diego, CA 92103-8890, USA; ^2^Department of Plastic, Wound and Burn Surgery, University of Kansas Medical Center, Kansas City, KS 66160, USA

## Abstract

The purpose of our study was to determine the factors that influence the use of telemedicine consultation by primary care physicians (PCPs) in the management of patients with problem wounds. A short questionnaire was administered to thirty-six PCPs who referred to our Wound Care Program within one year. Participants were asked to rate the importance of specific concerns and benefits regarding the role of wound care surgical specialist (WCSS) and the use of telemedicine consults prior to possible face-to-face consultation. Sixty percent of respondents felt comfortable with telemedicine consultation based on recommendations alone. The total number of patients referred for telemedicine consult was 230, and face-to-face consultation with a WCSS was offered and arranged for 30% of patients. The perception of shared decision making, streamlining patient care, and an opportunity for followup were all highly ranked benefits. The majority of PCPs (93%) agreed that telemedicine wound care consult is a useful tool in their practice and would continue to use the telemedicine consult service.

## 1. Introduction

Chronic wounds continue to be an important and costly healthcare concern across all (home, ambulatory, and hospital) settings [1]. Hospitalized, immobile patients with multiple medical problems are prone to develop nonhealing wounds. Frequently, there is difficulty in understanding and implementing wound preventive measures, management plans, and accessing wound care specialists [2]. Preliminary studies have shown that outreach wound care programs augmented by the store-and-forward type of telemedicine consultations are accurate and efficacious measures, allowing access to appropriate specialists for patients in rural and urban settings. Telemedicine consultation has also been shown to reduce overall costs, and decrease transportation issues and the time needed for implementing wound management plans [3–5]. The telemedicine-based feedback from the prospective consultant was well received by patients [6]. Telemedicine was found to be a valuable tool in providing useful preliminary information in acute situations requiring the referral of patients to plastic and reconstructive surgeons [7]. However, there is relatively little information on how using telemedicine may specifically increase effectiveness of the primary care physicians (PCPs) caring for patients with problematic wounds, improve collaboration with wound care surgical specialists (WCSSs), and impact PCPs' attitude towards telemedicine as an aid to practice [8, 9]. Therefore, the purpose of our study was to evaluate PCPs attitudes towards telemedicine and determine PCPs satisfaction from telemedicine consults for patients with problematic wounds.

## 2. Materials and Methods

A six-question survey (see the appendix) was prepared focusing on the factors that affect PCPs management of patients with the problem of chronic wounds in the setting of available telemedicine consultations by the local ambulatory Wound Care Program. The questions were developed based on discussions with PCP peers involved in wound care. Issues identified from other studies that surveyed PCPs satisfaction related to telemedicine services were also considered [8–10]. PCP's were asked to use a four-point scale, indicating the level of concern for each of several factors related to the telemedicine consultation by a WCSS or the level of helpfulness for several actions that could be recommended (or taken) by a WCSS. They were then asked to rank the top three of these factors or actions. Other questions included the appropriateness of transferring wound care from a WCSS to a PCP, the use of a multidisciplinary clinic and team for collaborative wound management, satisfaction from WCSS, and personal comfort in caring for chronic, difficult wounds. The survey was administered to 36 PCPs (Internal Medicine and Family Practice specialists) managing patients in long-term care and skilled nursing facilities, in Southern California, who referred at least 6 new patients to the ambulatory outreach Wound Care Program within one year (2007). Problem wounds were defined as those that did not heal for at least 6 weeks from the day of injury or wound diagnosis and commencement of the initial treatment (not under the auspices of the wound care specialist). Demographic parameters and wound epidemiology of patients were reviewed and tabulated (Table 1). There were no specific inclusion/exclusion patient criteria for a telemedicine consult, the order for an Outreach Wound Care Program and telemedicine consult by a wound care surgical specialist was solely a PCP decision. Patients referred for wound consultation were seen for the first time within 24 hours by the Wound Care Program Nurse. The nurse obtained the history, assessed the patient, and obtained wound data including standardized photographs and provided this to the WCSS (Board-Certified Plastic Surgeon) for assessment and formulation of the management proposal. Consultation statement included synopsis and discussion of findings, diagnosis, diagnostic workup (if needed) and treatment plan recommendations. These were then forwarded via protected email to the PCP who then had the option to adopt and enforce consult suggestions [5].

## 3. Results

A total of thirty six PCPs met the appropriate inclusion criteria mentioned previously and were emailed surveys. Thirty completed surveys were returned (83%). Six respondents practiced in rural settings, the remaining practiced in an urban area. The total number of patients referred for telemedicine consult was 230, and subsequently, 60 of these patients (26 percent) underwent surgical intervention. Sixty percent of PCPs (18 of 30) felt comfortable with telemedicine consultation and implementing recommendations of the initial management plan. In this group, there were no requests for subsequent direct consultation with WCSS or another surgical specialist unless requested by the WCSS. Amongst the group of PCPs with previous experience with the ambulatory Wound Care Program (12 of 30 physicians, 40%), the rate of acceptance of the telemedicine-based initial wound management recommendations was even higher. In this subgroup 9 of 12 PCPs (75%) declared “comfort with endorsing telemedicine consult derived recommendations”. 

Direct examination was requested by the PCP if uncomfortable with implementing the initial telemedicine consult plan or by the WCSS if further examination was felt to determine the overall care plan. A total of 69 (30%) referrals for direct examination were requested. In 18 of these 69 (26%) patients the WCSS suggested direct face-to-face examination. In the remaining 51 patients requests for direct examination was made by the PCP. These originated from 12 physicians (40%) who defined their level of confidence or comfort with telemedicine consult as the sole base of the development of the management plan, as neutral or limited. All patients suggested for direct examination by the WCSS, were seen within two weeks for direct consultation. No significant changes in the wound management plan (as delineated by telemedicine consult) were proposed after direct consultations [5]. PCPs were offered follow-up Wound Care Program nurse visit and subsequent telemedicine consults on a monthly and as needed basis. No PCP declared total lack of confidence in telemedicine wound consultation. Overall, a vast majority of PCPs (28 of 30, 93%) agreed that the telemedicine wound care consult is a useful, practice effectiveness enhancing tool, regardless whether they were willing to implement consultant recommendations without modifications or whether they treated consultant suggestions merely as a peripheral or intermediate modality. 

**Figure 1:**
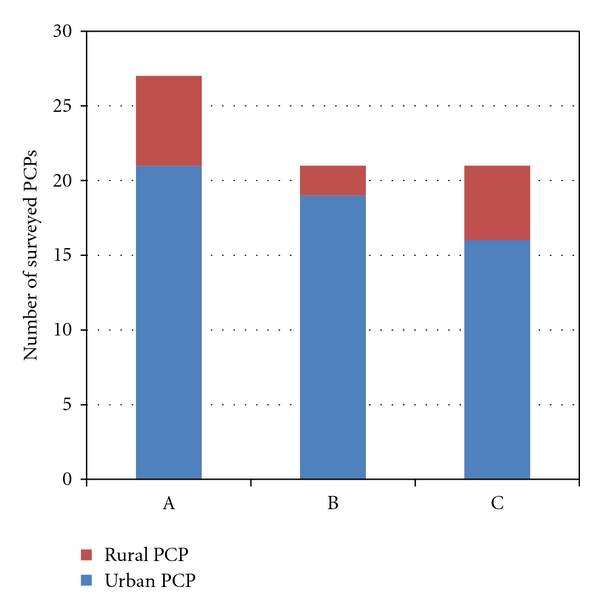
The three most important concerns regarding the telemedicine consult as rated by PCP. A: concern regarding the ability to reduce decisional uncertainty by the WCSS. B: concern regarding the ability to rely on telemedicine consult should the wound be unstable or worsen. C: concern regarding potential for medico legal risks because of decisions made based on telemedicine consultation.

**Figure 2:**
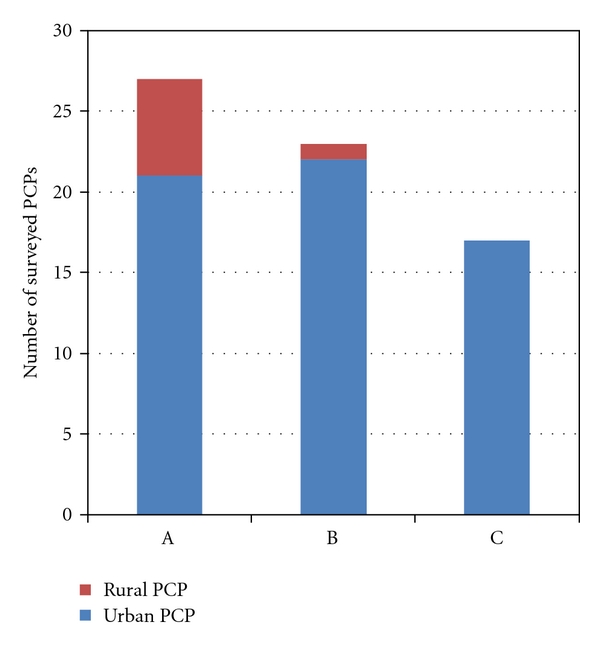
Telemedicine consults by WCSS deemed to be most valuable factors for PCPs. A: reduction of level of uncertainty regarding directing a patient to conservative treatment versus surgical intervention. B: patient and family perception of shared decision making. C: allowed for expeditious care of difficult wounds.

The decisional conflict defined as a state of uncertainty about the course of action to take (conservative versus surgical wound management) was reduced once the telemedicine consultation and wound management decision plan was offered to PCPs. This affirmation was ranked as number one PCP personal benefit from telemedicine consultation by a WCSS (as well as number one concern). Ranking of factors deemed most concerning regarding the telemedicine consultation for a problem wound by a WCSS and actions and/or recommendations by a WCSS deemed most helpful for a PCP were recorded. The three most important concerns regarding the telemedicine consult as rated by PCPs were (1) concern regarding the ability to reduce decisional uncertainty by the WCSS; (2) concern regarding the ability to rely on telemedicine consult should the wound worsen and (3) concern regarding potential for medico legal risks because of decisions made based on telemedicine consultation (Figure 1). The three most important benefits of telemedicine consultation rated by PCPs were (1) reduction of level of uncertainty regarding directing a patient to conservative treatment versus surgical intervention; (2) patient and family perception of shared decision making, and (3) efficient and expeditious care of difficult wounds (Figure 2). A Fifteen of the 30 PCPs (50%) stated that they felt that their bond with the patient and caretakers strengthened after telemedicine consultation with a WCSS.

## 4. Discussion

The management of patients with chronic wounds make up a significant portion of PCPs' practices [2–4]. The problematic wound may create concerns among PCPs [11]. Frequent uncertainty among PCPs about the course of action regarding wounds exemplifies need for effective, consumer-friendly decisions aiding in tripartite (patient, PCP and WCSS) communication support system [3, 5, 12]. The importance of decisional PCP conflict is demonstrated by both high ranking of this issue as a concern whether telemedicine was going to help as well as the most significant benefit after exposure to the program. The concern regarding the effectiveness of telemedicine to affirm the PCP wound management plan and ability to dissipate uncertainties was the leading preexisting concern, while effective reduction of decisional conflicts (conservative versus surgical management) was reported as the leading benefit after exposure to telemedicine.

No doubt that telemedicine facilitates the ability of a primary care physician to link with specialists such as WCSS [3, 5, 6, 13]. Interestingly, “more expeditious care” was identified as one of the leading benefits of telemedicine, however, there was no striking perception that outcomes would improve as well.

For patients the introduction of telemedicine consultation, prior to or replacing the face to face consult, seemed to educate patients, foster the sense of security, and create a bond with the consultant. It has been concluded that the telemedicine feedback is a useful tool in increasing patient comfort, enhancing perception that the surgical specialist is familiar with their condition, able and willing to control the management process, and involving them in the decision making process. Noteworthy is the higher rate of satisfaction and less uncertainty regarding the recommended course of care in patients subjected to telemedicine than in those that were not offered this service [6]. Patients seem to value increased frequency of contacts with providers even if it is via telemedicine encounters, more than actual lengthy “face-to-face” consultations. It appears that patients value encounters when they feel that physicians are focused on their problems and that their overall care is well monitored [6, 14].

The goal of this survey was to determine if WCSS are a useful tool with PCPs and what degree they meet the needs by providing telemedicine consultations? Surveyed PCPs perceived that telemedicine consultation and advice regarding problematic wound management only increased patient's desire to continue care under his or her direction and that specialist involvement did not erode their bond with patients. PCPs concerns, in other similar studies (not specifically related to wound care) relate to the possibility that the use of telemedicine may result in increased medical/surgical interventions recommended by specialists who may have less intimate knowledge of intricacies of their patient health, and other concerns about telemedicine relate to the effect it may have in eroding the involvement of PCPs in the care of their patients [15]. This concern has not emerged as the top listing in this study (listed agree only by 3, 10% of PCPs). Likely, both the increased perception of shared decisions making and improvement of expeditiousness of care (both listed by 70% of respondents) contributed to patient satisfaction and indirectly to PCPs perception that their bond with patients was enhanced by introduction of telemedicine wound consult. The opportunity for the followup, repeated telemedicine consultations was ranked the high (20 of 30, 60% of respondents) among program benefits. Overall, a vast majority of PCP (28 of 30, 93%) agreed that the telemedicine wound care consult is a useful, enhancing communication with the WCSS tool in their practice and would like to continue to use the telemedicine consult service.

Several survey questions had overlapping meaning. Responses to questions and comments strongly suggest that clarification of pros and cons of possible surgical intervention for a problem wound was the most important issue, because surgical option has a central and the most “dramatic” role in conversations between the PCP and patient when considering the diverse array of available management strategies for problematic wound. Thus, it appears that the PCP valued more directive rather than diagnostic confirmatory aspect of telemedicine consults by WCSS. Consequently, it can be concluded that in case of problem wounds the focus on managerial issues by WCSS addresses needs of PCPs [16]. Interestingly, PCP's felt more comfortable with managing patients wounds conservatively, although effective utilization of nonsurgical modalities requires sophisticated wound care knowledge [17].

Perhaps, increasing level of confidence and satisfaction rate from telemedicine consultations by a WCSS among those with previous experience with telemedicine services results in higher diagnostic accuracy, and reassurance that patients can be easily followed to discuss contingencies or even transfer of care can be arranged based on telemedicine consultation alone [4, 5].

## 5. Conclusion

Increased overall problem wound managerial confidence by PCPs due to telemedicine consults coinciding with positive patient perceptions is key for the Outreach Program and telemedicine success. The high rate of acceptance by PCPs and the overall positive feedback regarding telemedicine aided consults by a Wound Care Surgical Specialist verifies the program success as a relatively new, efficient, clinical tool in dealing with problem wounds.

## Figures and Tables

**Table 1:** Demographic parameters and wound epidemiology.

Patient characteristics	
Age (years), range 37 to 78 years, mean (±SD)	53.9 (±10.4)
Men/Women	117/113

Etiology of wounds	

Pressure sore	98
Venostasis ulcers	48
Arterial ulcers no diabetes	29
Diabetic foot	28
Radiation wounds	14
“Unstable” scars	5
Lymphoedema ulcers	3
Ulcerated skin cancer	3
Nonhealed old burns	2

## Appendix

We are studying factors that affect the problematic wound management by primary care physicians. We are specifically interested in patients who have been referred to a surgical wound care specialist for evaluation with the telemedicine consultation prior to possible face-to-face consultation. The patient is now returning to the care of their primary care physician. Recognizing that problem wounds can be a concern, we will concentrate on those patients whose management includes the primary care provider continuing management of their wounds.

1. Generally, all patients referred to a WCSS for a consultation should be managed by the specialist and wound care should be transferred.
  Agree
  Neutral to moderately agree
  Disagree
  Strongly disagree
2. Generally, as a PCP I feel more comfortable with managing wounds conservatively than deciding regarding surgical intervention.
  Yes
  No
3. Have you had any personal experience with telemedicine consultations prior to referral of patients to WCSS that this survey refers to?
  Yes
  No
4. How much do the following factors concern you in managing problematic wounds? Circle the number to the right to indicate level of concern then, please rank the top three most important factors by writing no. 1, no. 2, and no. 3 to the left of the statement.
5. Level of concern: 4 = very, agree or comfortable, 3 = moderately or neutral 2 = disagree or limited 1 = no concern, strongly disagree or lack of confidences.

Overall telemedicine was useful in your decision making process regarding wound management - - - - (4 3 2 1)
Telemedicine will reduce the level of your uncertainty regarding optimal management of the wound - - - -  (4 3 2 1)
Telemedicine-based input should help to understand the rationale behind the WCSS recommendations - - - -  (4 3 2 1)
It was face-to-face consultation that increased critically my understanding of the rationale behind the WCSS recommendations (if applicable) - - - -  (4 3 2 1)
Did telemedicine help you to deal with patient concern should the wound condition worsen - - - -  (4 3 2 1)
There is a high potential that a WCSS will take over patient care and proceed with interventions with no or little input of PCP - - - -  (4 3 2 1)
You felt comfortable in endorsing WCSS recommendations based on telemedicine consult alone - - - -  (4 3 2 1)
Face-to-face consultation was absolutely necessary following the telemedicine consult - - - -  (4 3 2 1)
I did not hesitate to refer my patient for face-to-face consultation when WCSS requested - - - -  (4 3 2 1)
There is an inherent problem with the diagnostic accuracy of telemedicine consults by WCSS - - - -  (4 3 2 1)
I feel comfortable enough with my WCSS telemedicine wounds assessment that I will let him/her to decide if my patients need to be evaluated also by a multidisciplinary Wound Care Center team - - - -  (4 3 2 1)
I am satisfied with WCSS telemedicine-based recommendations however, I miss more closer collaborative contacts - - - -  (4 3 2 1)
Quick telemedicine wound consults and the availability of follow up consults help me to manage concurrent patients problems - - - -  (4 3 2 1)
For patients undergoing only conservative wound care telemedicine consults are valuable in terms of understanding modalities available for conservative wound management - - - -  (4 3 2 1)
Qualified WCSS may provide equally effective consultation regarding the initial management of problematic wound as the multidisciplinary team - - - -  (4 3 2 1)
There is a potential for unusual medicolegal problems because of telemedicine-based consultation - - - -  (4 3 2 1)
I am overall satisfied from telemedicine use in wound care - - - -  (4 3 2 1)

(5) How helpful would these factors be in allowing you to become more comfortable with continuing patient wound care in a situation that the wound is not responsive to treatment or the condition worsens? Rank the top three most important factors by writing no. 1, no. 2, and no. 3 to the left of the statement.
Importance: 4 = helpful, 3 = somewhat helpful, 2 = neutral, 1 = not needed

Telemedicine reduced the level of my uncertainty regarding wound workup and management plan - - - -  (4 3 2 1)
Thanks to telemedicine, I will know earlier when my patient requires surgical intervention - - - -  (4 3 2 1)
Improved coordination of wound care with WCSS before the patient returns to care directly managed by PCP in the form of personal discussion - - - -  (4 3 2 1)
Explicit written instructions (alternative protocols) from WCSS via telemedicine rather than conversation - - - -  (4 3 2 1)
Ability to do phone follow-up consults with WCSS when problem(s) arise - - - -  (4 3 2 1)
Assurance that the option of transfer of care to WCSS will always exist - - - -  (4 3 2 1)
Patient and family perception of shared decision making - - - -  (4 3 2 1)
Positive feedback from patients and perception of improved PCP relationship with wound care patients subjected to telemedicine interaction with WCSS - - - -  (4 3 2 1)
Access to the newest information regarding wound care through WCSS resources - - - -  (4 3 2 1)
Thanks to telemedicine, I provided more expeditious care - - - -  (4 3 2 1)
Thanks to telemedicine, service wound care outcomes in my practice will improve - - - -  (4 3 2 1)

(6) Background information: (please circle the answer)
  Sex: M F
  Degree: M.D., D.O.
  Years in clinical practice: < than 5 5–10 10–15 15–20 >20
  Residency completed: No Yes
  Type of current practice: solo single specialty group multispecialty group
  Practice setting: urban rural
  Number of providers in the group: 1 2–5 5–10 10–20 >20
  Have you referred patients to a WCSS within the past year: 1–6 6–12 >12.
